# Vascularized in vitro bone model as 3D quadruple culture with primary human osteoblasts, osteocytes, osteoclasts and endothelial cells

**DOI:** 10.1016/j.mtbio.2025.102154

**Published:** 2025-08-05

**Authors:** Katharina Wirsig, Nina Bürger, Lisa Fleischhauer, Nele Louisa Preuß, Anne Bernhardt

**Affiliations:** Centre for Translational Bone, Joint- and Soft Tissue Research, Faculty of Medicine and University Hospital, TUD University of Technology, Fetscherstraße 74, 01307, Dresden, Germany

**Keywords:** In vitro bone model, Osteoblasts, Osteocytes, Osteoclasts, Endothelial cells, Co-culture, Quadruple culture

## Abstract

With an aging population worldwide, research into bone metabolism and novel therapies for damaged and diseased bone is essential. Bone is a vascularized, dynamic tissue that undergoes a constant remodeling process mediated by osteocytes, osteoblasts and osteoclasts. In this study, a complex 3D in vitro bone model combining these three main bone cell species with endothelial cells was developed. The different cell species were isolated from primary human tissue and spatially arranged using transwell inserts. Osteocytes differentiated from collagen-embedded osteoblasts, while osteoclasts simultaneously derived from peripheral blood mononuclear cells without receptor activator of NF-κB ligand (RANKL) supplementation. Different cultivation parameters were evaluated to define conditions that support differentiation and function of all cell types involved in quadruple culture. The cellular crosstalk in quadruple cultures stimulated osteoblast (ALP, BMP-2, IBSP, COL1A, VEGF) and osteocyte (SOST, DMP1) markers, while osteoclast (TRAP) and endothelial cell markers were reduced compared to respective mono- or co-cultures. Furthermore, mineralization was induced only in quadruple cultures, demonstrating the importance of signaling between the four cell types. This sophisticated human bone model provides a physiologically relevant culture system to study the complex crosstalk between bone cells, their precursors and endothelial cells during remodeling and vascularization. Moreover, it allows preclinical testing of bioactive factors, biomaterial extracts or drugs for translation into clinical practice without animal testing.

## Highlights

• Simultaneous differentiation of osteocytes and osteoclasts without RANKL supplementation.
• Osteogenesis, osteoclastogenesis, vascularization and mineralization in parallel.
• Osteocyte differentiation is upregulated by BMP-2 (MEPE, SOST, DMP1).
• Crosstalk in quadruple culture stimulated osteoblast markers and mineralization.
• Crosstalk in quadruple culture stimulated osteocyte markers (SOST, DMP1).

## Graphical abstract

**Figure:**
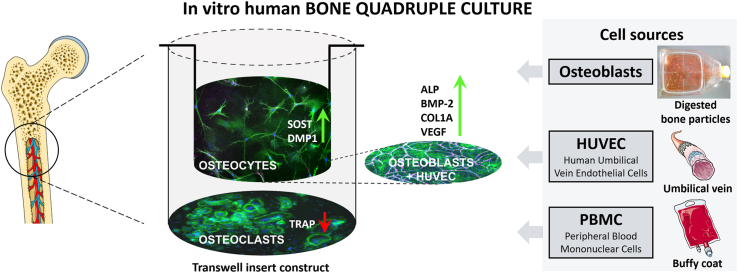

## 1 Introduction

Bone tissue undergoes a cycle of continuous bone formation and degradation known as bone remodeling. This dynamic process is enabled by the coordinated activity of bone cells and their crosstalk. Osteocytes (OCy), embedded in the bone matrix, are the most abundant cell type in bone tissue and orchestrate the function of bone-forming osteoblasts (OB) and bone-resorbing osteoclasts (OC) [1,2]. Derived from OB, OCy act as mechanosensors and initiate the cellular response toward bone formation or degradation. The secretion of sclerostin (SOST), a critical marker for OCy, regulates the production of receptor activator of NF-κB ligand (RANKL) in OCy [3]. Thus, OCy and OB, which secrete RANKL and its antagonist osteoprotegerin (OPG) [4], directly influence the differentiation of OC and their resorptive activity. The interaction of RANKL with its receptor RANK, located on the surface of OC progenitors, stimulates the fusion of monocytes into multinucleated, resorptive OC. OPG can bind to RANKL preventing the interaction with RANK, thereby inhibiting the differentiation and activity of OC [5]. This RANK/RANKL/OPG mechanism underscores the fundamental role of crosstalk between different bone cell types in maintaining bone homeostasis and regulating bone formation and resorption. Imbalances in bone homeostasis can lead to severe disorders such as osteoporosis, characterized by decreased bone density due to excessive OC activity and impaired bone formation, or osteopetrosis, marked by abnormal bone formation resulting in overly dense bone tissue.

Bone is a highly vascularized tissue with an extensive network of blood vessels ensuring supply of oxygen and nutrients. Endothelial cells (EC) form the inner layer of blood vessels, and their proliferation is a prerequisite for angiogenesis, the formation of new blood vessels [6]. During bone remodeling, angiogenesis is crutial to deliver factors necessary for bone formation demonstrating the importance of EC for bone healing and regeneration [7]. In turn, bone cells produce signals that influence EC. Both OB and OCy secrete vascular endothelial growth factor (VEGF) which promotes angiogenesis in areas of bone remodeling or repair [8,9]. Hypoxic stimuli induce the expression of VEGF in OB by hypoxia-inducible factor-α (HIF-α) family [10,11]. In addition, angiopoietins and Notch signaling are identified as modulators of both angiogenesis [12] and bone remodeling [13,14]. Notch signaling in EC is known to influence osteogenic differentiation of OB by increasing bone morphogenetic protein 2 (BMP-2), which is involved in the mineralization process [15]. On the other hand, Notch is part of the RANKL/OPG regulation [16], which is essential for bone resorption. In summary, the interaction between bone cells and EC is critical for the maintenance of bone health, including efficient bone remodeling, maintenance of bone strength and the repair of damaged bone.

Therefore, in vitro models that recapitulate bone metabolism and remodeling should include both, bone cell types and EC. The primary advantage and interest of in vitro models is the reduction of animal testing, which raises ethical concerns, is expensive, time-consuming and does not necessarily represent the mechanisms and responses in the human organism. In vitro bone models frequently use cell lines instead of primary cells, such as murine OCy-like MLO-Y4 cells [9,17], murine OB-like MC3T3 [18,19]/human MG-63 cells [20,21] or murine OC progenitor-like RAW 264.7 cells [22]. In addition to the cell line characteristics, which exhibit differences in cellular behavior compared to primary cells, these bone cell lines are mostly derived from animals, such as mice, rather than humans, or are tumor-related, such as Saos-2 cells [23]. Although some in vitro bone models comprise primary human cells, such as co-cultures with OB and OC progenitors [24] or co-cultures with OB and OCy [25], those with multiple cell types are rare [26]. We have recently developed a bone triple culture with primary human OB and simultaneously differentiating OCy and OC [27]. In this model, the supplementation of triple culture medium with RANKL was not necessary for OC differentiation from peripheral blood mononuclear cells (PBMC), because OB and OCy in the triple culture secreted RANKL, which stimulated OC differentiation. Thus, this model is suitable for studying bone metabolism, including differentiation processes and cellular crosstalk. Since the aforementioned bone model lacks EC, we focused here on a vascularized in vitro bone model as quadruple culture with primary human OB, OCy, OC and EC. Therefore, commercially available and widely used human umbilical vein endothelial cells (HUVEC) were utilized. Bongio et al. published a bone tetra-culture comprising HUVEC, bone marrow mesenchymal stem cells, OB and OC precursors embedded in a collagen/fibrin hydrogel [28]. However, the different cell types were mixed in the hydrogel, preventing separate analysis of the cell types. To address this limitation, in the present study, three-dimensional quadruple cultures were assembled with transwell inserts to spatially separate the cell types for later analysis while allowing their crosstalk. To recapitulate the hierarchical architecture of the bone tissue, OCy were differentiated in a collagen gel, serving as three dimensional environment because OCy are deeply embedded in the bone matrix in vivo. In contrast, OB and OC are located on the bone surface in vivo and are therefore applied as two-dimensional cell layers in quadruple cultures. Only OB and HUVEC were cultured in direct contact, as EC require OB support to form tube-like structures. To develop this bone model, first, conditions must be found that support the simultaneous survival, function and differentiation of the three bone cell types and HUVEC. Second, cells in quadruple cultures were compared to respective cell types in monocultures (OB, OCy), if possible, or in co-cultures with supporting OB (OB-OC, OB-HUVEC) to investigate the impact of cellular crosstalk in the in vitro bone models.

## 2 Materials and methods

### 2.1 Cell culture

Primary human pre-OB were isolated from femoral heads of osteoarthritic patients undergoing total hip replacement or from knee femoral condyles during total knee joint replacement (arthroplasty) at the University Hospital *Carl Gustav Carus* Dresden (Germany) after informed consent (approval by the ethics commission of TU Dresden, EK 303082014 and BO-EK-235052022) as previously described [29]. Pre-OB were then expanded in α-MEM with GlutaMAX (Gibco) containing 10 % FCS, 100 U/mL penicillin and 100 μg/mL streptomycin (PS) (Gibco) until passage 4 (Table 1). Primary OB were routinely tested for mycoplasma.

**Table 1:** Media for quadruple cultures experiments. Fetal calf serum (FCS), penicillin/streptomycin (PS), endothelial cell growth medium (EGM), β-glycerophosphate (β-GP), ascorbic acid-2-phosphate (AAP), dexamethasone (Dex), heat inactivated FCS (hi FCS), macrophage colony stimulating factor (MCSF), endothelial cell basal medium (ECBM), endothelial cell growth medium (EGM), vascular endothelial growth factor (VEGF), bone morphogenetic protein 2 (BMP-2).

Purpose	Oxygen level	Media composition
**Expansion OB**	Normoxia (21 % O_2_)	10 % FCS, 1 % PS in α-MEM
**Expansion HUVEC**	Normoxia	EGM (Promocell)
**Osteogenic differentiation OB**	Normoxia	**Expansion OB** + 10 mM β-GP, 12.5 μg/mL AAP, 10^−7^ M Dex
**Expansion PBMC**	Normoxia	10 % hi FCS, 25 ng/mL MCSF, 1 % PS in α-MEM
Basal medium quadruple culture **(BM quadruple)**	–	2 % hi FCS, 5 mM β-GP, 12.5 μg/mL AAP, 1 % PS in 50 % α-MEM/50 % ECBM
**Quadruple culture**	Normoxia	**BM quadruple** + 20 ng/mL VEGF
		**BM quadruple** + 20 ng/mL VEGF + 100 ng/mL BMP-2
	Hypoxia (5 % O_2_)	**BM quadruple**
		**BM quadruple** + 100 ng/mL BMP-2

For osteogenic differentiation, human pre-OB in passage 4 were cultured in osteogenic medium (expansion medium supplemented with 10^−7^ M dexamethasone (Dex), 10 mM β-glycerophosphate (β-GP) and 12.5 μg/mL ascorbic acid-2-phosphate (AAP), all osteogenic supplements from Sigma-Aldrich) for 7 days. For OB monoculture, 1•10^4^ OB per well in 48-well plates were seeded in expansion medium. After three days, medium was changed to quadruple culture medium (Table 1) and OB were cultivated for 14 days, with a medium change after seven days.

After osteogenic differentiation, OB were harvested with Trypsin/EDTA (Gibco) and embedded in collagen gels for differentiation of OCy, as previously published [30]. Briefly, eight parts of collagen solution (4 mg/mL rat tail collagen, Meidrix Biomedicals GmbH in 0.1 M acetic acid) and one part of 10x HBSS, were neutralized with 1 N NaOH and supplemented with 10 mM β-GP and 12.5 μg/mL AAP. OB were resuspended in a concentration of 1•10^5^ cells/mL. For OCy monocultures, 500 μL of the collagen/cell suspension was pipetted into 24-well plates. After 30 min at 37 °C, collagen was fibrillated into a stable hydrogel and quadruple culture medium was added (Table 1). OB in collagen gels were incubated for 14 days with a medium change after seven days to complete OCy differentiation.

HUVEC, purchased from Promocell, were expanded in endothelial cell growth medium (EGM, Promocell) until passage 3. For OB-HUVEC co-cultures, OB after osteogenic differentiation were seeded in expansion medium (1•10^4^ per well in 48-well plates). After three days, HUVEC in passage 4 were seeded on top of OB (1•10^4^ per well) and medium was changed to quadruple culture medium (Table 1). OB-HUVEC co-cultures were conducted over 14 days with a medium change after seven days.

OC were differentiated from PBMC, isolated from human buffy coat concentrates (purchased from German Red Cross Dresden) by density gradient centrifugation as previously published [31]. For OB-OC co-cultures, 1.5•10^6^ PBMC per 24-well were seeded in PBMC expansion medium, containing 25 ng/mL MCSF (Table 1). After three days, pre-differentiated OB were seeded in 24-well-transwell inserts (1•10^4^ OB per insert), inserted to the well plates with pre-seeded PBMC and quadruple culture medium (Table 1) was added. PBMC differentiate into OC over 14 days in co-culture with OBs with a medium change after 7 days.

### 2.2 Quadruple cultures

**Fig. 1:**
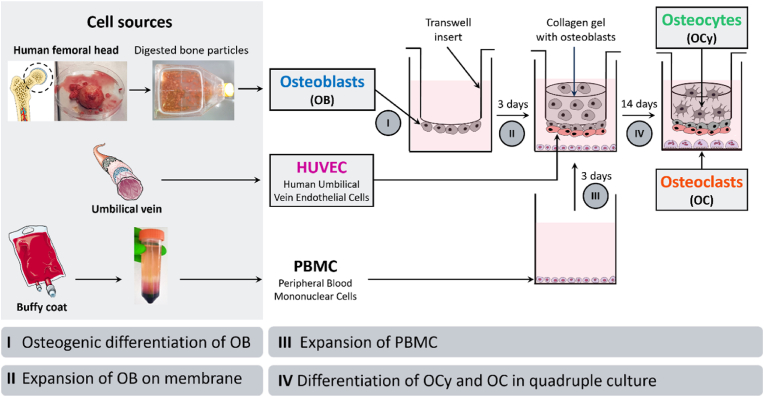
Experimental setup for bone quadruple cultures with primary human OB, OCy, OC and endothelial cells. OB were isolated from human femoral heads or femoral condyle. PBMC were isolated from human buffy coat by density gradient centrifugation. HUVEC, on top of OB were seeded to the apical side of transwell insert membranes. OB embedded in collagen gel were transferred to the basal side of the transwell inserts. Constructs were placed in well plates with PBMC. Over 14 days, collagen gel embedded OB differentiate to OCys and OCs differentiate simultaneously from PBMC.

Bone quadruple cultures with primary human OB, OCy, OC and EC were assembled using transwell inserts (0.4 μm pore size, Sarstedt) in 24-well plates (Fig. 1). OCy differentiate from collagen gel embedded OB and OC differentiate synchronously from PBMC in quadruple cultures. First, pre-differentiated OB were seeded to the apical side of transwell insert membranes (1•10^4^ per insert membrane) and were allowed to attach to the polyethylene terephthalate (PET) membrane for 1 h at 37 °C without further medium supply. Subsequently, OB expansion medium was added and OB were incubated at 37 °C. After three days, HUVEC (1•10^4^ per insert membrane) were seeded on top of OB and allowed to attach for 30 min. Afterwards, OB embedded in collagen gel (same collagen solution and cell density as for OCy monocultures) were transferred to the basal side of the transwell inserts. For collagen gelation, samples were incubated at 37 °C for 30 min. Constructs were then placed in 24-well plates, pre-seeded with PBMC (three days before). PBMC had been incubated in PBMC expansion medium until assembly of quadruple cultures. After building up quadruple cultures, medium was added (Table 1). Quadruple culture medium is based on 50 % α-MEM (Gibco) and 50 % endothelial cell basal medium (ECBM; Promocell). Because a low serum content is favorable for OCy differentiation [32] and heat inactivated (hi) FCS is preferred for differentiation of OC, the basic quadruple culture medium contains 2 % hi FCS. In addition, osteogenic supplements β-GP and AAP as well as PS were added. The two main factors investigated to ascertain conditions for a bone quadruple culture were the oxygen level and the presence of BMP-2 (recombinant human BMP-2/InductOs; Medtronic) (Table 2). Under normoxic conditions (21 % O_2_), quadruple cultures received VEGF (recombinant human VEGF_165_; PeproTech) for survival and tube formation of HUVEC. Under hypoxia (5 % O_2_), no external VEGF was added because we hypothesized stimulated VEGF production by OB and OCy under low oxygen levels. Under both conditions, cells were cultured with and without BMP-2, as BMP-2 has been shown to be essential for stable OCy differentiation [27]. Bone quadruple cultures were maintained at 37 °C in an incubator with adjustable O_2_ and CO_2_ concentrations for 14 days, with a medium change after 7 days.

**Table 2:** Experimental groups and donor information of four individual quadruple culture experiments with different donor combinations.

Aim	Exp. Nr.	Donor combination	Donor information hOB/hOCy	Conditions	Samples
**Conditions for Quadruple culture**	Exp.1	hOB 1 hOCy 1 HUVEC 1 PBMC 1	Male 65 years	Normoxia + VEGF	8x **Quadruple** culture per condition
				Normoxia + VEGF + BMP-2	
				Hypoxia	
				Hypoxia + BMP-2	
	Exp.2	hOB 2 hOCy 2 HUVEC 1 PBMC 1	Female 62 years	Normoxia + VEGF	8x **Quadruple** culture per condition
				Normoxia + VEGF + BMP-2	
				Hypoxia	
				Hypoxia + BMP-2	
**Quadruple culture VS** **Mono-/Co-cultures**	Exp.3	hOB 3 hOCy 3 HUVEC 2 PBMC 2	Female 62 years	Normoxia + VEGF + BMP-2	8x **Quadruple** culture +8x **mono** hOB 3 +8x **mono** hOCy 3 +8x **Co** hOB 3-HUVEC 2 +8x **Co** hOB 3-PBMC 2
	Exp.4	hOB 3 hOCy 3 HUVEC 2 PBMC 3	Female 62 years	Normoxia + VEGF + BMP-2	8x **Quadruple** culture +8x **mono** hOB 3 +8x **mono** hOCy 3 +8x **Co** hOB 3-HUVEC 2 +8x **Co** hOB 3-PBMC 3

Quadruple cultures were analysed in four independent experiments with different donor combinations, involving OB/OCy of three different donors, HUVEC of two donors and PBMC of three donors (Table 2). Information on age and sex of HUVEC and PBMC was not provided.

### 2.3 Fluorescence microscopy

Transwell insert membranes with OB + HUVEC were separated from transwell inserts using surgical knife and tweezers. OCy in collagen gels were removed from transwell inserts. Membranes with OB + HUVEC, OCy in collagen gels and OC in well plates were washed (PBS), fixed (4 % formaldehyde in PBS), permeabilised (0.1 % Triton X-100 in PBS for 5 min, followed by washing with PBS) and blocked (1 % BSA in PBS for 30 min).

OB + HUVEC were incubated with 1 μg/mL mouse anti-human CD31 (Dako; Clone JC70A) and OCy were incubated with either 3.3 μg/mL rabbit anti-human DMP1 (TaKaRa; M176), 100 μg/mL rabbit anti-human SOST (antibodies-online GmbH; ABIN1714867) or 1 μg/mL mouse anti-human PDPN (antibodies-online GmbH; ABIN6939085) over night and after washing incubated with 1 μg/mL DAPI, Phalloidin-iFluor 488 (Abcam) as well as 4 μg/mL AlexaFluor 546 goat anti-mouse IgG (Invitrogen) for CD31 and PDPN or 8 μg/mL AlexaFluor 546 goat anti-rabbit IgG (Invitrogen) for DMP1 and SOST. OB cultures were incubated with DAPI/Phalliodin-iFluor488 accordingly. Z-stack images were captured using a Keyence BZ-X810 fluorescence microscope. Image processing was performed with ImageJ (Fiji).

### 2.4 RNA isolation, cDNA synthesis and PCR

PET membranes with OB + HUVEC were cut of the transwell inserts using surgical knife and tweezers. Collagen gels with OCy were removed from inserts and digested in collagenase II solution (3 mg/mL collagenase II in α-MEM, 10 % FCS, 3 mM CaCl_2_, and 1 % PS for 1 h at 37 °C. Digests were washed with PBS and centrifuged to obtain OCy pellets. Four quadruple culture samples of each group were used to isolate RNA of membranes with OB + HUVEC, OCy pellets and OC in well plates using the commercially available peqGOLD MicroSpin Total RNA Kit (Peqlab). Lysates of two samples were pooled to finally obtain two RNA samples per group.

For cDNA synthesis, the High-Capacity cDNA Reverse Transcription Kit (Applied Biosystems) was applied according to the manufacturer’s instructions.

**Fig. 2:**
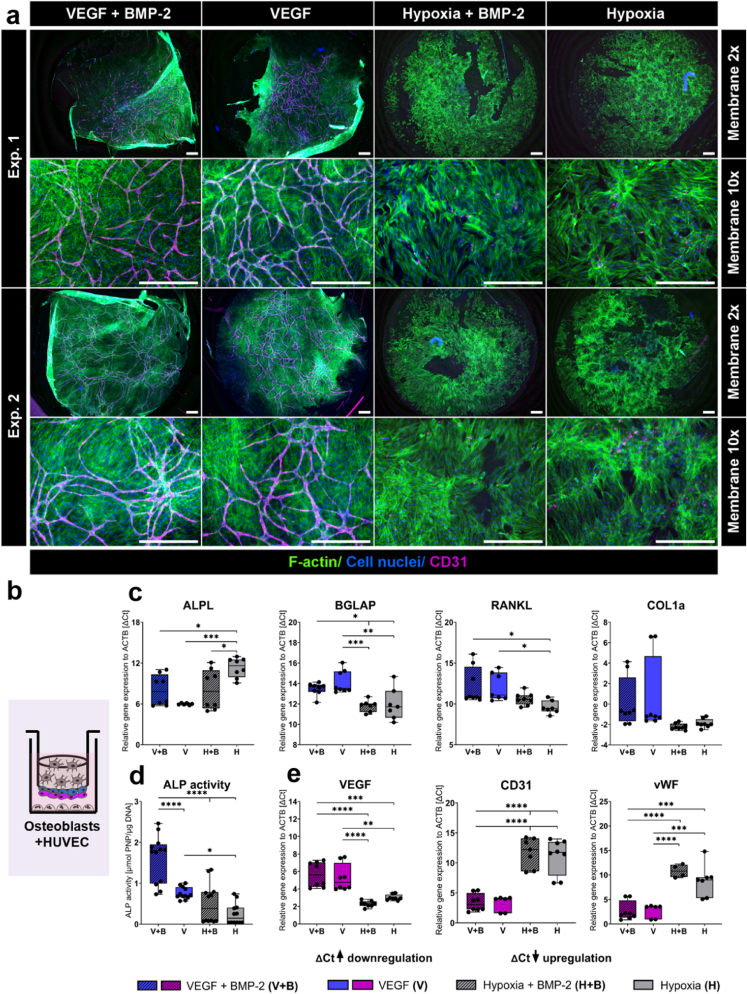
**OB and HUVEC on transwell insert membranes in two quadruple culture experiments under different cultivation conditions.** (a) Fluorescence microscopic images. Cytoskeleton appears green (iFluor488 phalloidin), nuclei appear blue (DAPI) and CD31 appears magenta (Alexa Fluor 546). Scale bars represent 500 μm. (b) Scheme of OB + HUVEC in quadruple culture. (c) Gene expression of OB markers presented as ΔCt (each experiment *n* = 4, in total *n* = 8). (d) ALP activity of OB (each experiment *n* = 3, in total *n* = 6). (e) Gene expression of endothelial cell markers presented as ΔCt (each experiment *n* = 4, in total *n* = 8). ∗p < 0.05; ∗∗p < 0.01; ∗∗∗p < 0.001; ∗∗∗∗p < 0.0001. (For interpretation of the references to colour in this figure legend, the reader is referred to the Web version of this article.)

**Fig. 3:**
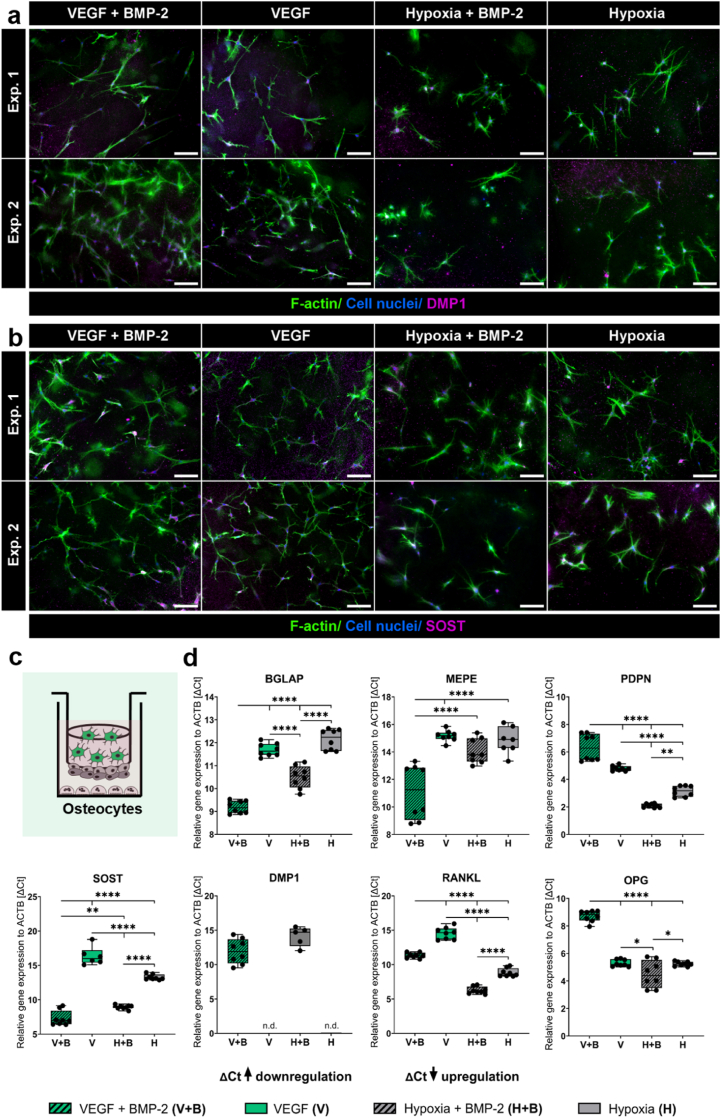
**OCy in two quadruple culture experiments under different cultivation conditions.** (a, b) Fluorescence microscopic images. Cytoskeleton appears green (iFluor488 phalloidin), nuclei appear blue (DAPI) and DMP1 (a)/SOST (b) appear magenta (Alexa Fluor 546). Scale bars represent 100 μm. (c) Scheme of OCy in quadruple culture. (d) Gene expression of OCy markers presented as ΔCt (each experiment *n* = 4, in total *n* = 8). ∗p < 0.05; ∗∗p < 0.01; ∗∗∗∗p < 0.0001. (For interpretation of the references to colour in this figure legend, the reader is referred to the Web version of this article.)

**Fig. 4:**
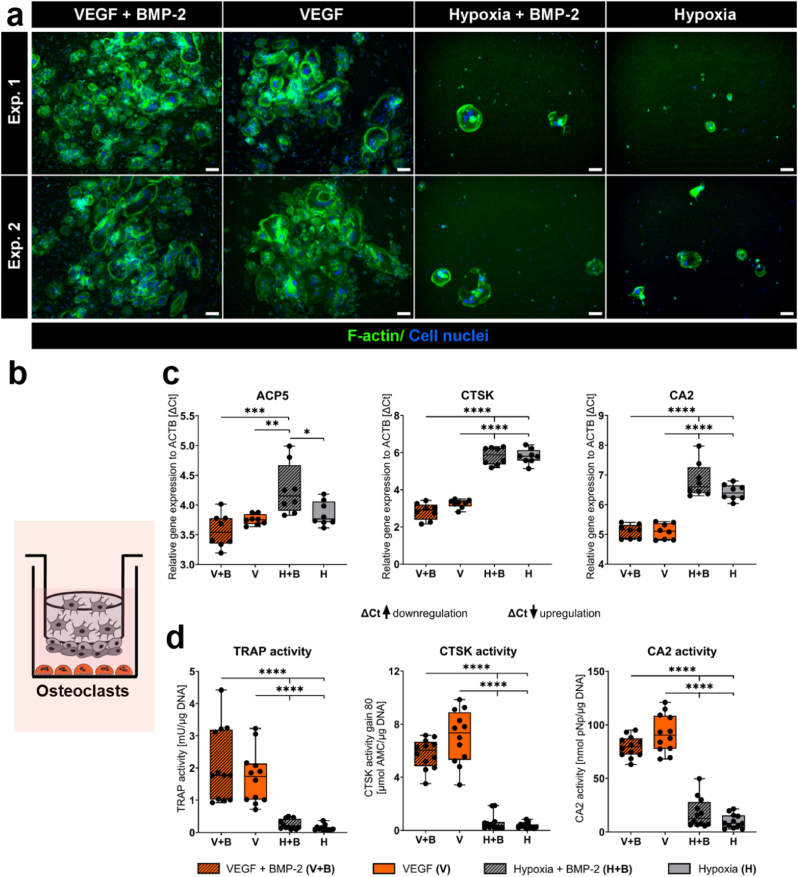
**OC in two quadruple culture experiments under different cultivation conditions.** (a) Fluorescence microscopic images. Cytoskeleton appears green (iFluor488 phalloidin), nuclei appear blue (DAPI). Scale bars represent 100 μm. (b) Scheme of OC in quadruple culture. (c) Gene expression of OC markers presented as ΔCt (each experiment *n* = 4, in total *n* = 8). (d) TRAP, CTSK and CA2 actvity (each experiment *n* = 3, in total *n* = 6). ∗p < 0.05; ∗∗p < 0.01; ∗∗∗p < 0.001; ∗∗∗∗p < 0.0001. (For interpretation of the references to colour in this figure legend, the reader is referred to the Web version of this article.)

**Fig. 5:**
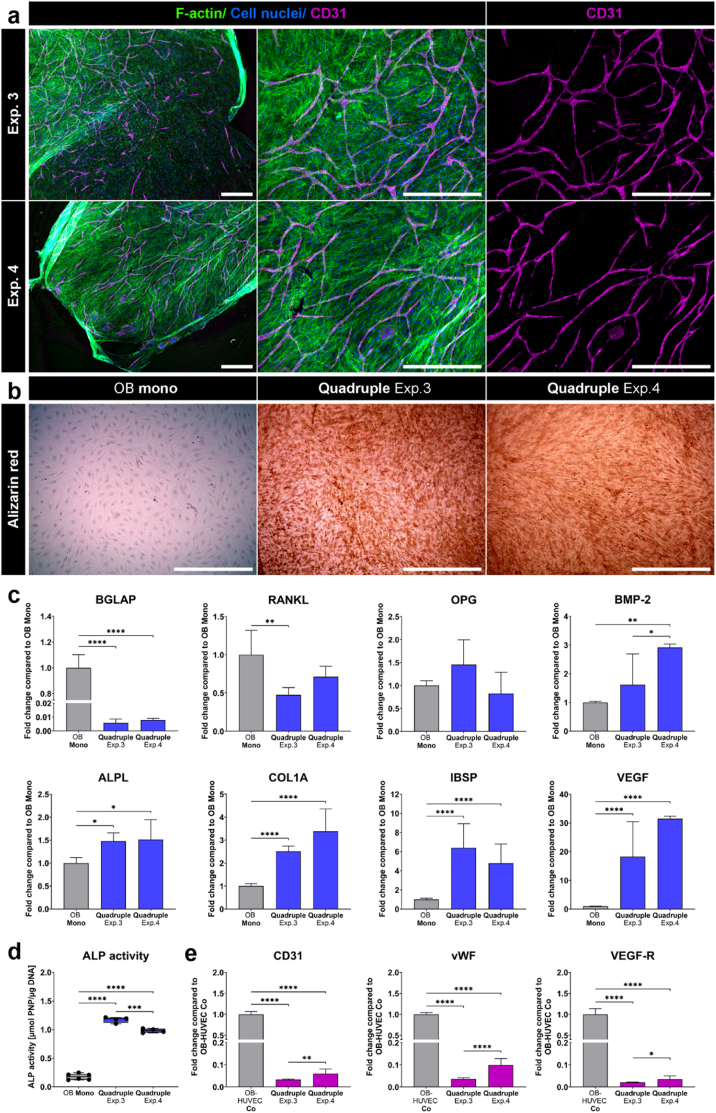
**OB and HUVEC on transwell insert membranes in two quadruple culture experiments in comparison to OB monocultures or OB-HUVEC co-cultures.** (a) Fluorescence microscopic images in quadruple culture. Cytoskeleton appears green (iFluor488 phalloidin), nuclei appear blue (DAPI) and CD31 appears magenta (Alexa Fluor 546). Scale bars represent 500 μm. (b) Alizarin red staining of OB in monoculture and quadruple culture. Scale bars represent 500 μm. (c) Gene expression of OB markers presented as fold change ± upper and lower limit normalized to OB monocultures (each experiment *n* = 4). (d) ALP activity of OB (each experiment *n* = 3). (e) Gene expression of endothelial cell markers presented as fold change ± upper and lower limit normalized to OB-HUVEC co-cultures (each experiment *n* = 4). ∗p < 0.05; ∗∗p < 0.01; ∗∗∗p < 0.001; ∗∗∗∗p < 0.0001. (For interpretation of the references to colour in this figure legend, the reader is referred to the Web version of this article.)

**Fig. 6:**
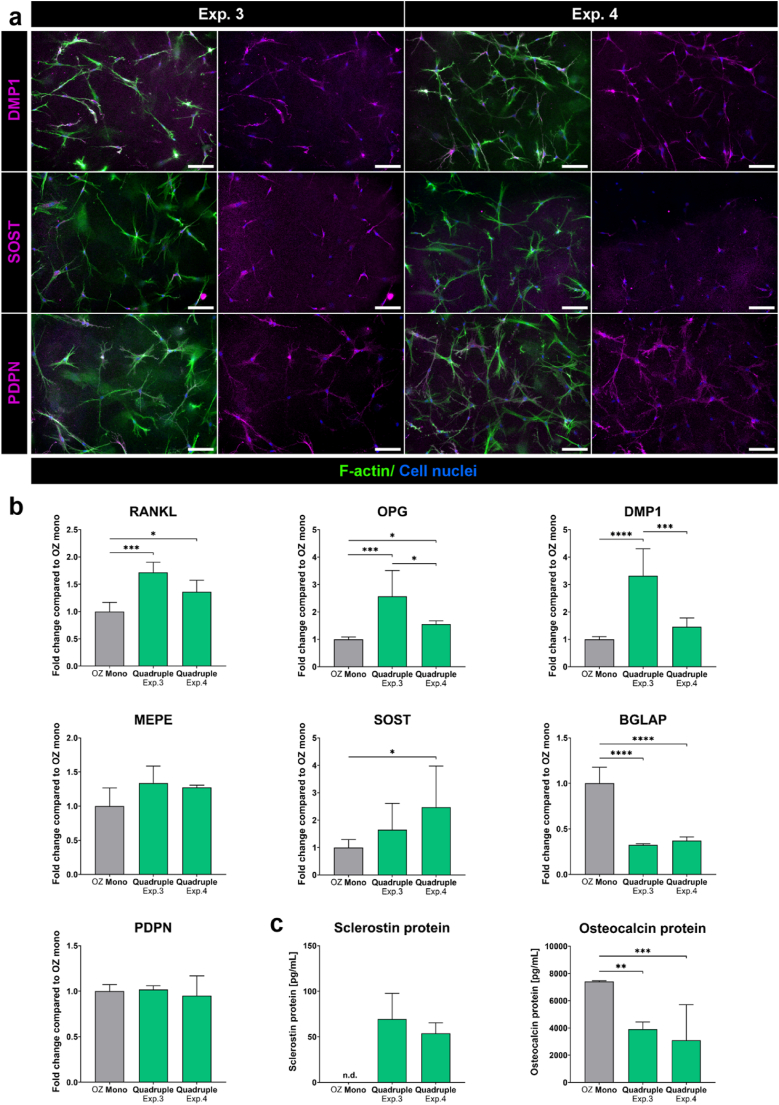
**OCy in two quadruple culture experiments in comparison to OCy monocultures.** (a) Fluorescence microscopic images in quadruple culture. Cytoskeleton appears green (iFluor488 phalloidin), nuclei appear blue (DAPI) and DMP1/SOST/PDPN appear magenta (Alexa Fluor 546). Scale bars represent 100 μm. (b) Gene expression of OCy markers presented as fold change ± upper and lower limit normalized to OCy monocultures (each experiment *n* = 4). (c) Secreted BGLAP and SOST protein in cell culture supernatants (each experiment *n* = 3). ∗p < 0.05; ∗∗p < 0.01; ∗∗∗p < 0.001; ∗∗∗∗p < 0.0001. Not detectable (n.d.). (For interpretation of the references to colour in this figure legend, the reader is referred to the Web version of this article.)

**Fig. 7:**
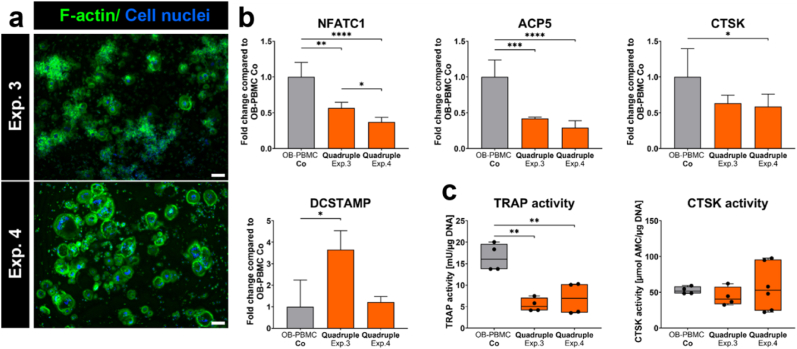
**OC in two quadruple culture experiments in comparison to OB-OC co-cultures.** (a) Fluorescence microscopic images in quadruple culture. Cytoskeleton appears green (iFluor488 phalloidin) and nuclei appear blue (DAPI). Scale bars represent 100 μm. (b) Gene expression of OC markers presented as fold change ± upper and lower limit normalized to OB-OC co-cultures (each experiment *n* = 4). (c) OC-specific enzyme activities (each experiment *n* = 3). ∗p < 0.05; ∗∗p < 0.01; ∗∗∗p < 0.001; ∗∗∗∗p < 0.0001. (For interpretation of the references to colour in this figure legend, the reader is referred to the Web version of this article.)

Gene expression analysis was performed using qPCR reactions with the TaqMan Fast Advanced Master Mix (Applied Biosystems) and TaqMan Gene Expression Assays (all Applied Biosystems) for the following genes: actin-β (ACTB; Hs01060665_g1), alkaline phosphatase (ALPL; Hs01029144_m1), bone gamma-carboxyglutamate protein (osteocalcin/BGLAP; Hs01587814_g1), bone sialoprotein II (BSP II, IBSP; Hs00173720_m1), receptor activator of NF-κB ligand (RANKL/TNFSF11; Hs00243522_m1), osteoprotegerin (OPG/TNFRSF11B; Hs00900358_m1), podoplanin (PDPN; Hs00366766_m1), matrix extracellular phosphoglycoprotein (MEPE; Hs00220237_m1), dentin matrix protein 1 (DMP1; Hs01009391_g1), sclerostin (SOST; Hs00228830_m1), collagen type I alpha 1 chain (COL1A; Hs00164004_m1), bone morphogenetic protein 2 (BMP-2; Hs00154192_m1), platelet endothelial cell adhesion molecule (PECAM1/CD31; Hs01065279_m1), vascular endothelial growth factor (VEGF; Hs00900055_m1), vascular endothelial growth factor receptor 2/kinase insert domain receptor (VEGFR/KDR; Hs00911700_m1), von willebrand factor (vWF; Hs01109446_m1), tartrate-resistant acid phosphatase (ACP5/TRAP; Hs00356261_m1), cathepsin K (CTSK; Hs00166156_m1) and carbonic anhydrase II (CA2; Hs01070108_m1), nuclear factor of activated T-cells cytoplasmic (NFATC1; Hs00542675_m1), dendrocyte expressed seven transmembrane protein (DCSTAMP; Hs00229255_m1), according to manufacturer’s instructions. PCR was run with an Applied Biosystems 7500 fast Real-Time PCR system. Gene expression was normalized to the expression of ACTB (ΔCt) and relative gene expression (fold change) was calculated using the 2^−ΔΔCt^ method. Gene expression data in Fig. 2, Fig. 3, Fig. 4 are presented as ΔCt values to quantitatively visualize marker gene expression and to define best conditions for quadruple cultures. Lower ΔCt values indicate strong expression, while higher ΔCt values indicate lower expression. Fold changes were calculated in Fig. 5, Fig. 6, Fig. 7 and normalized to the previously defined control quadruple culture medium (VEGF + BMP-2) to qualitatively represent gene expression in quadruple cultures compared to mono/co-cultures.

### 2.5 Alizarin red staining

Alizarin red staining was applied for detection of calcium, indicating mineralization. Therefore, transwell insert membranes with OB + HUVEC of quadruple cultures or OB in monoculture in 48 well plates after 14 days cultivation were fixed with 4 % formaldehyde in PBS for 1 h and, after washing with distilled water, incubated in 2 % alizarin red dissolved in distilled water (pH 4.1–4.3; Sigma Aldrich) for 3 min. After washing with distilled water, brightfield images were captured using a Keyence BZ-X810 microscope. Image processing was performed using ImageJ (Fiji).

### 2.6 Quantification of specific enzyme activities

ALP activity of OB monocultures and OB + HUVEC on transwell insert membranes in quadruple cultures as well as TRAP, CTSK and CA2 activity of OC in quadruple culture and OB-PBMC co-cultures were measured and normalized to the DNA content of the respective samples. Samples were thawed and lysed with 1 % Triton X-100 in PBS for 50 min with an ultrasonication step for 10 min in between. ALP activity was determined colorimetrically by the cleavage of colourless p-nitrophenyl phosphate to yellowish p-nitrophenol and quantified by absorbance measurements at 405 nm [33].

Osteoclast specific enzyme activities were quantified as previously published [34]. Briefly, CA2 activity was determined calorimetrically by the cleavage of colourless p-nitrophenyl acetate into yellowish p-nitrophenol and quantified by absorbance measurements at 405 nm. TRAP activity was measured by cleavage of naphthol ASBI phosphate at acidic pH in the presence of tartrate and detection of fluorescence signals at an excitation and emission wavelength of 405/520 nm. CTSK activity was quantified by the cleavage of Z-LR-AMC (Enzo Life sciences) with fluorescence measurements at an excitation and emission wavelength of 365/440 nm. For all absorbance measurements a spectrofluorometer infinite M200pro (Tecan Trading AG, Switzerland) was used.

DNA concentration of cell lysates was quantified using Quantifluor One dsDNA kit (Promega) according to manufacturer’s instructions.

### 2.7 Osteocalcin and sclerostin ELISA

Cell culture supernatants were analysed with the following ELISA kits: Human Osteocalcin DuoSet ELISA # DY1419-05 and Human SOST/Sclerostin DuoSet ELISA # DY1406, both R&D Systems, using recombinant human BGLAP (312–10000 pg/mL) or SOST (62.5–4000 pg/mL) calibration curves.

### 2.8 Statistics

Quadruple cultures with OB, OCy, OC and HUVEC were performed in four independent experiments with different donor combinations (Table 2). For each quadruple culture condition, monoculture or co-culture, samples of the individual experiments were seeded in triplicates for the measurement of cell type specific enzyme activities and ELISA-based quantification of protein secretion in cell culture supernatant, while gene expression was analysed in two RNA samples generated from four replicates for each experimental group. Statistical differences in gene expression were calculated at the level of ΔCt values. Normal (Gaussian) distribution of all data was confirmed by Shapiro-Wilk normality test. One-way ANOVA, followed by Tukeys’s multiple comparisons test was used to compare the experimental groups. GraphPad Prism 10.0 was used for all statistical analyses.

## 3 Results

In an advanced in vitro bone model as quadruple culture, it is essential to find a compromise for cultivation conditions that support OB, OCy, OC and HUVEC in parallel. Therefore, different media compositions (with and without VEGF and BMP-2) as well as normoxic and mild hypoxic cultivation were tested in two quadruple culture experiments with different donor combinations (Exp.1, Exp. 2; Table 1, Table 2) with respect to their impact on these four cell types. Hypoxic cultivation was hypothesized to stimulate VEGF and BMP-2 production of OB and OCy instead of external supplementation.

### 3.1 Normoxic cultivation with VEGF supplementation allows network-formation of HUVEC in quadruple culture

After 14 days in quadruple culture, OB covered the entire transwell insert membrane in all examined groups. Only with VEGF supplementation under normoxic cultivation, HUVEC on top of OB (Fig. 2b) formed a dense network of tube-like structures, visualized in magenta by CD31 staining (Fig. 2a). In contrast, hypoxic cultivation, regardless of BMP-2 supplementation, resulted in loss of HUVEC and no network-formation. OB-specific ALPL expression (p < 0.001) and corresponding ALP activity (p < 0.0001) were reduced under hypoxia compared to normoxic cultivation (Fig. 2c and d). BMP-2 treatment induced ALPL expression under hypoxia (p < 0.05) and ALP activity under normoxia (p < 0.0001). Furthermore, BGLAP and RANKL gene expression were significantly increased under hypoxia compared to normoxic cultivation with VEGF (Fig. 2c). Hypoxia strongly stimulated VEGF expression, but reduced CD31 and vWF expression of HUVEC (Fig. 2e).

### 3.2 BMP-2 stimulates OCy differentiation in quadruple culture

OB embedded in collagen gel developed OCy-specific three-dimensional dendritic processes in all tested groups and showed a positive staining for DMP1 in the center of the cells under normoxic conditions and a positive staining for SOST in all conditions, but weaker in hypoxia compared to normoxia (Fig. 3a–c). BMP-2 treatment strongly stimulated expression of OCy-marker genes BGLAP, SOST and RANKL (all p < 0.0001) (Fig. 3c). In addition, DMP1 expression was only detectable in the presence of BMP-2. Gene expression of MEPE induced with BMP-2 supplementation under normoxic conditions (p < 0.0001) and PDPN was upregulated by BMP-2 under hypoxia (p < 0.01) compared to BMP-2-free medium. Hypoxic cultivation with 5 % O_2_ led to a significant reduction in gene expression of BGLAP (p < 0.0001) and MEPE, while PDPN (p < 0.0001) and RANKL (p < 0.0001) were strongly induced compared to normoxic cultivation. Furthermore, OPG gene expression increased under hypoxia in the presence of BMP-2 compared to BMP-2-free cultivation (p < 0.0001).

### 3.3 Mild hypoxia inhibits OC formation

Multinucleated, roundish OC differentiated from PBMC in quadruple culture under normoxic conditions in the presence and absence of BMP-2 (Fig. 4a and b). Fluorescence images of OC with separated channels for cytoskeleton and nuclei are shown in the supplementary material to clearly demonstrate the multinucleated character of OC in quadruple cultures (Fig. S1). No direct effect of BMP-2 on OC-formation was detactable. Hypoxic cultivation under 5 % O_2_ significantly inhibited OC-formation, with very few OC-like cells observed regardless of BMP-2 supplementation. Moreover, OC-specific TRAP/ACP5, CTSK (p < 0.0001) and CA2 (p < 0.001) gene expression, along with corresponding enzyme activities (all p < 0.0001) were strongly decreased under reduced oxygen levels compared to normoxic cultivation of quadruple cultures (Fig. 4c and d). BMP-2 treatment did not cause changes in OC-specific enzyme activities.

With respect to 3.1.-3.3., for further quadruple culture experiments with OB, OCy, OC and HUVEC, normoxic cultivation with VEGF and BMP-2 supplementation of the quadruple culture medium was chosen to ensure stable OCy differentiation by BMP-2, HUVEC network-formation by VEGF and OC-formation by normoxic cultivation. In the following experiments (Exp.3, Exp.4, Table 1, Table 2), cell types in quadruple culture were compared with respective cell types in monocultures (OB, OCy), if possible, or in co-cultures with supporting OB (OB-OC, OB-HUVEC) in the defined quadruple culture medium to investigate the relevance of cellular crosstalk in a bone in vitro model.

### 3.4 ALP, BMP-2, COL1A, IBSP, VEGF and mineralization are stimulated in OB in quadruple culture

In line with findings presented in section 3.1., HUVEC on OB in quadruple culture migrated directionally and established a branched network of tube-like structures in normoxia in the presence of VEGF (Fig. 5a). Furthermore, BGLAP (p < 0.0001) and RANKL (p < 0.01) gene expression was reduced in OB + HUVEC in quadruple culture compared to OB monocultures (Fig. 5c). Conversely, BMP-2 (p < 0.01), COL1A, IBSP and VEGF (all p < 0.0001) were strongly induced in quadruple culture. In addition, ALPL gene expression and ALP activity of OB were significantly increased in quadruple culture compared to OB monoculture (Fig. 5c and d). Furthermore, mineralization occurred only in quadruple culture, not in OB monoculture (Fig. 5b). EC marker genes of HUVEC (CD31, vWF and VEGF-R) were detected in quadruple culture; however, these genes were significantly reduced in the presence of OCy and OC in quadruple culture compared to OB-HUVEC co-cultures (Fig. 5e).

### 3.5 DMP1, SOST, RANKL and OPG are induced in OCy in quadruple culture

OB, embedded in collagen gel, differentiated over 14 days in quadruple culture into DMP1, SOST and PDPN expressing OCy (Fig. 6a and b). In addition, RANKL, OPG and DMP1 gene expression increased in quadruple culture, with significant differences compared to OCy monocultures, while PDPN and MEPE were not significantly affected (Fig. 6b). Furthermore, SOST gene expression and SOST protein concentration in cell culture supernatants were strongly induced in quadruple culture (Fig. 6b and c). In contrast, BGLAP gene expression and protein levels were reduced.

### 3.6 OC marker expression of NFATC1, ACP5 and CTSK is reduced in quadruple culture

PBMC differentiated into multinucleated OC in quadruple culture (Fig. 7a). Gene expression analysis revealed the presence of the OC marker genes NFATC1, ACP5, CTSK and DCSTAMP. However, the expression of these genes, with exception of DCSTAMP, were significantly reduced in quadruple cultures compared to OB-OC co-cultures (Fig. 7b). Concurrently, TRAP activity of OC was decreased in quadruple cultures (Fig. 7c).

### 3.7 OB, OCy, OC and HUVEC strongly express their marker genes in quadruple culture

After 14 days in quadruple culture, expression of cell type-specific marker genes of OB, OCy, OC and endothelial cells was detected in four independent experiments with different donor combinations in quadruple culture medium supplemented with VEGF and BMP-2 (Fig. 2, Fig. 3, Fig. 4, Fig. 5, Fig. 6, Fig. 7). These cultivation parameters were defined as control condition for further application of the bone quadruple culture as in vitro test platform. Six of eight detected OB marker genes (ALPL, COL1A, VEGF, IBSP, BMP-2, OPG) were expressed in all experiments with ΔCt <15 (Supplementary Material Fig. S2a) under this conditions. In parallel, all four examined EC marker genes (CD31, vWF, VEGF-R, CD5) were strongly expressed (ΔCt <10; Fig. S2b). Furthermore, five out of seven analysed OCy marker genes (MEPE, RANKL, OPG, BGLAP, PDPN) were expressed with ΔCt <15 (Fig. S2c) and all five OC-markers (ACP5, CA2, CTSK, DCSTAMP, NFATC1) showed strong gene expression (dCt <10; Fig. S2d) too. The detection of low ΔCt values of marker genes for all cell types involved in quadruple culture proofed their strong expression, indicating successful differentiation of all cell types.

## 4 Discussion

To mimic bone metabolism in vitro for studying cellular signaling and differentiation processes, multiple cell types are required. In addition to bone cell types, including OCy, which mediate bone formation of OB and bone resorption of OC, EC are important in the cellular crosstalk that controls the bone remodeling process. We have recently established a bone triple culture of primary human OB and simultaneously differentiated OC and OCy [27]. A limitation of this bone model is the lack of EC, a cell species involved in angiogenesis, which is a prerequisite for new bone formation. In the present study, we aimed at a quadruple culture with primary human OB, OCy, OC and EC by introducing HUVEC to the aforementioned triple culture.

The introduction of an additional cell type to an already established co-culture model requires the evaluation of different cultivation conditions to support viability and differentiation of all involved cell types. In general, as few external differentiation factors as possible should be added to the common culture medium to allow the analysis of cellular crosstalk and differentiation processes by the signaling between different cell types. Therefore, mild hypoxic cultivation under 5 % O_2_ was tested to stimulate VEGF secretion of OB and OCy. VEGF is under the control of oxygen levels through the HIF-1α signaling pathway [35,36]. As expected, VEGF gene expression of OB was stimulated under hypoxia (Fig. 1e), but nevertheless, HUVEC were not able to survive or form network-structures under this condition. Dense network-formation of HUVEC in vitro typically requires the external supplementation of VEGF at concentrations of 20 ng/ml [37]. VEGF protein concentration in cell culture supernatant under hypoxic conditions in our study, was below 1 ng/ml (data not shown), therefore the effective concentration was not reached and is probably responsible for the loss of HUVEC in hypoxic groups. Accordingly, EC markers CD31 and vWF were suppressed under hypoxia and without external VEGF supplementation (Fig. 2). A decreased Ca^2+^ restoring ability of the endoplasmic reticulum under hypoxia might also contribute to the observed disruption of angiogenic function [38]. In addition, hypoxia induces the production of reactive oxygen species (ROS) that damage endothelial cells through the PI3K/stathmin1 pathway [[39], [40], [41]] and cause tissue damage, dysfunction or inflammation promoting catabolism of bone tissue [42]. On the side of OB in quadruple culture, ALP gene expression and activity were decreased under hypoxic conditions, as previously described [[43], [44], [45]] and related to a reduction of the transcription factor Runx2 [46,47]. In turn, increased ALP of OB under normoxic conditions could be attributed to the VEGF supplementation of the normoxic group, which stimulates osteogenic differentiation [[48], [49], [50]]. Furthermore, hypoxia stimulated RANKL of OB and OCy in quadruple culture (Fig. 2, Fig. 3), which was also observed in human periodontal ligament cells [51] and explained by a HIF1α-induced upregulation of MAPK signaling pathway [52]. In contrast, RANKL-mediated OC formation was inhibited in quadruple culture under hypoxia, and OC-specific TRAP, CA2, and CTSK gene expression as well as corresponding enzyme activities were reduced (Fig. 4), as reported before in triple culture with mature OC [44]. Increased interleukin-33 (IL-33) expression in OB after HIF1α activation could be responsible for the inhibition of osteoclastogenesis [53]. Additionally, disruption of osteoclastogenesis under constant hypoxia was associated with suppression of nuclear factor of activated T-cell cytoplasmic 1 (NFATc1) [54]. This dual effect of hypoxia on osteoclastogenesis, with induced expression of RANKL, the main stimulator of osteoclastogenesis, but inhibited OC formation from PBMC and reduced OC-specific enzyme activities, reflects the partially contradictory effects of hypoxia on OC reported earlier [54,55]. However, our findings suggest that hypoxia-induced inhibition of OC formation is independent of RANKL. Another stimulus for OC formation and activity under normoxia is the presence of VEGF, which directly enhances survival and bone resorption of mature OC [56,57]. Consistent with the present study, hypoxia decreased expression of BGLAP in OCy [44]. However, OB displayed increased BGLAP expression after hypoxic cultivation (Fig. 2). Hirao et al. reported induction of BGLAP in OB-like MC3T3-E1 cells after 14 days of hypoxic culture, but suppression of BGLAP at later time points after mineralization (day 35) [58]. These observations suggest activation of mineralization under hypoxia by OB, but not by mature OCy.

In a previous study, we found that BMP-2 is essential for stable OCy differentiation of collagen gel embedded OB, especially OCy markers SOST, DMP1 and MEPE [27]. Because hypoxic stimuli are able to activate BMP-2 production of OB [59], quadruple culture medium with and without BMP-2 supplementation was examined under hypoxia and normoxia. However, BMP-2 was not detectable in quadruple culture supernatant without external BMP-2 supplementation, regardless of oxygen levels. BMPs, especially BMP-2, induce osteogenic differentiation [60] including the expression of Runx2 and ALP in pre-OB [61] which was also observed in OB in quadruple culture. BMP-2 seems to stimulate ALP activity of OB by activation of Wnt signaling pathway [62,63]. In addition, RANKL expression of OCy was increased by BMP-2, demonstrating the impact of BMP-2 on both osteogenesis and osteoclastogenesis [61]. However, BMP-2 treatment neither promoted OC formation and activity nor impaired OC in quadruple culture (Fig. 4). OCy marker expression in quadruple culture (SOST, DMP1, MEPE, BGLAP, RANKL; Fig. 2) was strongly induced by BMP-2, as expected and observed before in triple culture [27]. BMP-2 treatment also stimulated SOST expression in MLO-5 osteocytes [64] and Saos-2 cells [65]. SOST has a bidirectional regulatory role in bone remodeling and is thought to be a key mediator between bone formation and resorption [66]. As an antagonist of Wnt/β-catenin signaling, SOST inhibits bone formation [67,68], but without directly interfering with BMP-2 induced activation of OB-specific ALP [68]. Accordingly, BMP-2 induced ALP activity in quadruple culture occurred in parallel with highly stimulated SOST expression of OCy. Furthermore, SOST supports OC formation and activity through a RANKL-dependent pathway, that regulates the dynamic RANKL/OPG ratio, leading to either bone formation or resorption [69]. Because of this regulatory role, the detection of SOST in an in vitro model to study bone remodeling is of great importance.

In summary, only normoxic cultivation of quadruple cultures with VEGF and BMP-2 supplementation fulfilled the prerequisite of parallel survival, differentiation, typical morphology, enzyme activities and marker gene expression of OB, OCy, OC and EC and was therefore selected as the quadruple culture medium for further experiments and also defined as the control condition for future studies testing various parameters such as the effect of bioactive substances with this bone model.

The comparison of respective bone cell types in mono/co-cultures and quadruple culture revealed major differences in marker gene expression and enzyme activity levels due to the crosstalk between different cell types in quadruple culture. OC need the support of OB-produced RANKL in co-culture for their differentiation from PBMC and EC need OB as feeder layer in a co-culture to form networks. Therefore, OC and EC in co-culture with OB were used as comparison to quadruple cultures. OB markers ALP, IBSP, BMP-2, COL1A and VEGF were significantly increased in the presence of OCy, OC and EC in quadruple culture compared to OB monocultures. Induction of ALP and IBSP of OB in the presence of OCy and OC was also reported by Bernhardt et al. [30] and partially referred to soluble OC-produced factors like triple helix repeat containing 1 (CTHRC1), complement component c (C3) or spingosin phosphate kinase, which stimulate osteogenic differentiation [70]. In addition, the interaction of EC and OB induces osteogenesis, an effect that is attributed with activation of the Notch signaling pathway [71]. Furthermore, ALP induction, mediated by EC is associated with p38 mitogen-activated protein kinase-dependent pathway [72], contributing to the observed ALP increase in quadruple culture. Increased COL1A in the presence of EC, as observed in quadruple culture, was published recently and connected to Notch signaling activation [73]. In turn, all EC marker were reduced in the presence of OCy and OC in quadruple culture, but network formation of HUVEC was not impaired. Contrary to the upregulation of the aforementioned osteogenic markers, BGLAP expression of OB and OCy as well as BGLAP protein concentration in quadruple culture supernatant was reduced compared to OB/OCy in monoculture. A previous study hypothesized an inhibiting effect of OC on BGLAP of OB, while the presence of OCy stimulated BGLAP [30]. OCy-specific DMP1 and SOST were stimulated in quadruple culture, indicating a direct effect of OB, OC and EC on the expression of late OCy markers. OC in quadruple culture showed a decrease of their marker expression and TRAP activity, compared to OB-OC co-cultures. Since the presence of OCy did not significantly change TRAP gene expression and activity of mature OC [30], the presence of OB and EC cells is obviously responsible for the decrease of OC markers in quadruple culture. However, OC differentiate successfully from PBMC in quadruple culture by signaling between the different cell types without the external addition of RANKL. Moreover, RANKL expression of OCy in quadruple culture was increased compared to OCy monocultures.

## 5 Conclusion

The established advanced in vitro bone model as quadruple culture with primary human OB, OCy, OC and EC demonstrated differentiation, morphology and marker gene expression of all four cell types as well as enzyme activities of OB and OC in parallel. In general, the complex interaction of cell types in quadruple culture, stimulated OB and OCy markers as well as mineralization, while OC and EC markers were reduced compared to simple mono or co-cultures. However, whereas other in vitro bone models often focus on specific aspects of bone biology, like the bone marrow niche [74] or the early bone metastatic niche [75], our highly sophisticated bone model is suitable for studying the mechanisms behind bone remodeling, including the crosstalk between OB, OCy, OC and EC involved in osteogenesis, osteoclastogenesis and vascularization. An advantage of the quadruple culture setup is the use of transwell inserts to spatially separate the cell types for later analysis, but allow the complex crosstalk by soluble signals or direct contact in case of OB and EC. To our knowledge, the quadruple culture described here is the first in vitro bone model comprising primary human OB, OCy, OC and EC. Other bone models containing multiple cell types exist, but separate analysis of the respective cell types is precluded by the mixed cultivation of HUVEC, bone marrow mesenchymal stem cells, OB and OC precursors embedded in a collagen/fibrin hydrogel [28] or embedded OB, OC, bone-resident macrophages, EC and breast cancer cells in a bone minitissue [76]. Furthermore, such more complex, multicellular in vitro models are required to more reliably recapitulate the bone microenvironment compared to simplified in vitro models. This is supported by studies showing significant differences in response to drugs such as doxorubicin [76] or bioactive substances like high sulfated hyaluronan [27] in simpler monocultures compared to multicellular models. In conclusion, the established quadruple culture is an advanced human bone in vitro model that allows studies of the complex crosstalk between bone cells and EC. This includes investigations of osteogenesis, OCy differentiation, osteoclastogenesis, mineralization and vascularization in parallel in a controlled environment with exclusively primary human cells. In addition, long-term cultivation for more than 14 days is easily possible if required and would allow further applications. Comparing cellular behavior in quadruple cultures at different time points could provide answers about cyclic changes in osteoblast and osteoclast activity, thereby recapitulating the bone remodeling process. However, a large sample size is required for several experimental groups and time points. Therefore, the model’s main application is not high-throughput screening. Due to its complexity, time consumption, and resource requirements, the bone model can be used to answer more specific basic research question. For example, it can be used to test fewer pre-selected drug candidates or biomaterial extracts by comparing a control group (with the here defined cultivation conditions) to any treated group. Additionally, cellular crosstalk in response to changes in the cultivation environment could be analysed, such as changes in the mechanical environment of OCy in the collagen gel and how these changes affect the transduction of biochemical signals to the other cell types. Moreover, pathological bone conditions, such as inflammation, can be simulated by adding pro-inflammatory factors, such as IL-1β or TNF-α, to quadruple cultures. Comparing this to a control group would allow to draw conclusions about crosstalk between cells in quadruple culture under “inflamed” conditions. Furthermore, depending on the research question, the experimental setup allows for a broader range of analyses, such as more basic metabolic assays quantifying ATP or glucose, or the addition of a resorbable matrix to study osteoclast resorption. Taken together, the model is a suitable in vitro testing platform for drugs, bioactive substances or biomaterial extracts. It holds potential to reduce the need of animal testing in early stages of development and is preferable to existing in vitro bone models.

## CRediT authorship contribution statement

**Katharina Wirsig:** Writing – review & editing, Writing – original draft, Supervision, Methodology, Investigation, Formal analysis, Data curation, Conceptualization. **Nina Bürger:** Writing – review & editing, Methodology, Investigation, Formal analysis, Data curation. **Lisa Fleischhauer:** Writing – review & editing, Methodology, Investigation, Formal analysis, Data curation. **Nele Louisa Preuß:** Writing – review & editing, Methodology, Investigation, Formal analysis, Data curation. **Anne Bernhardt:** Writing – review & editing, Supervision, Resources, Project administration, Funding acquisition, Conceptualization.

## Declaration of competing interest

The authors declare that they have no known competing financial interests or personal relationships that could have appeared to influence the work reported in this paper.

## Data Availability

Data will be made available on request.
