# Supplementary material for: Vascularized in vitro bone model as 3D quadruple culture with primary human osteoblasts, osteocytes, osteoclasts and endothelial cells
Source: Mater Today Bio. 2025 Aug 5;34:102154. doi: 10.1016/j.mtbio.2025.102154 (PMC12345344; doi:10.1016/j.mtbio.2025.102154)
Supplement: Multimedia component 1 [file mmc1.docx]

**Supplementary material**

**Vascularized in vitro bone model as 3D quadruple culture with primary human osteoblasts, osteocytes, osteoclasts and endothelial cells**

Katharina Wirsig, Nina Bürger, Lisa Fleischhauer, Nele L. Preuß, Anne Bernhardt*


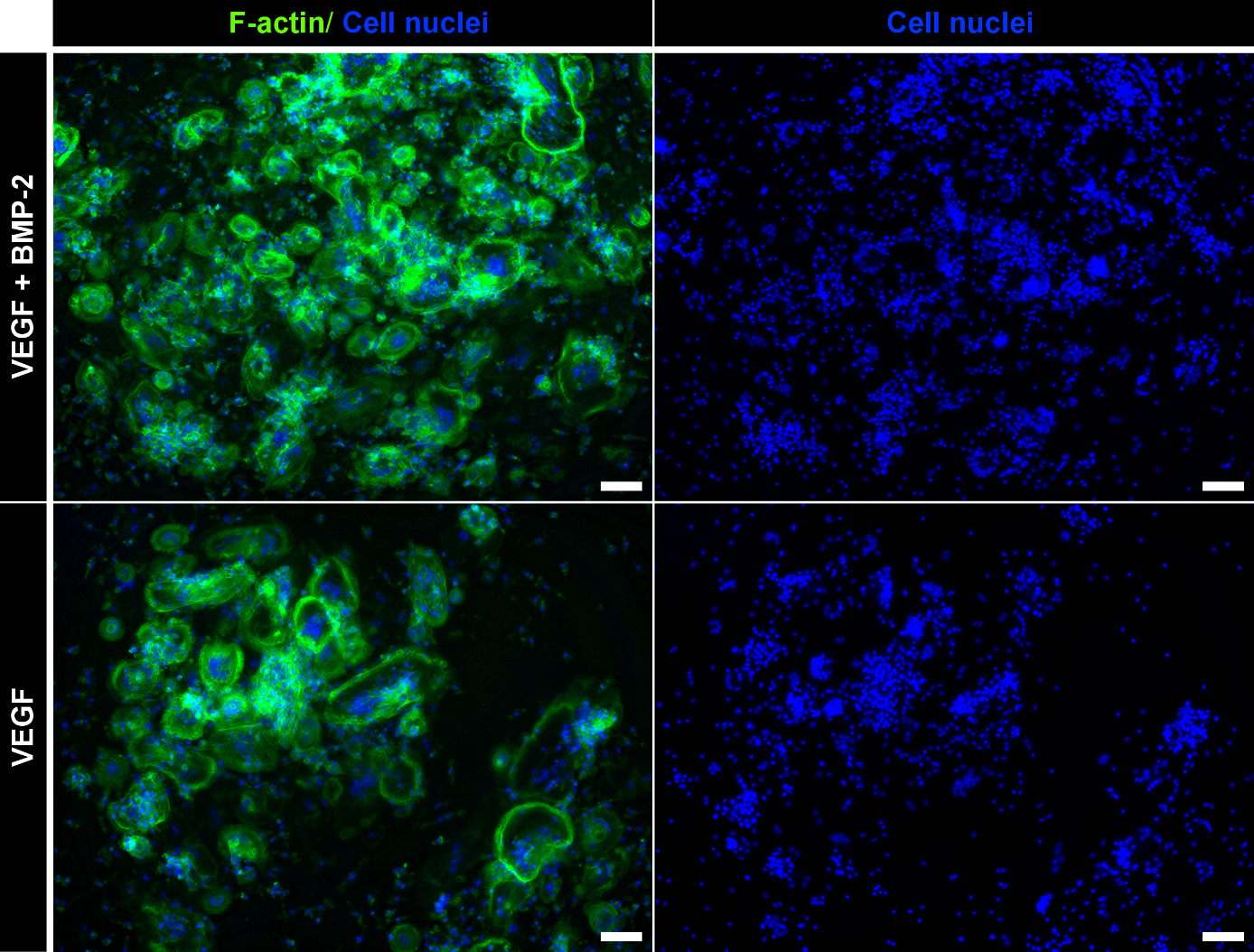


**Figure S1:** Enlarged fluorescene microscopic images of OC in quadruple culture Exp.1 after normoxic cultivation with VEGF + BMP-2 or only VEGF supplementation of the quadruple culture medium. Multinuclearity of differentiated OC is clearly visible. Cytoskeleton appears green (iFluor488 phalloidin) and nuclei appear blue (DAPI). Scale bars represent 100 µm.


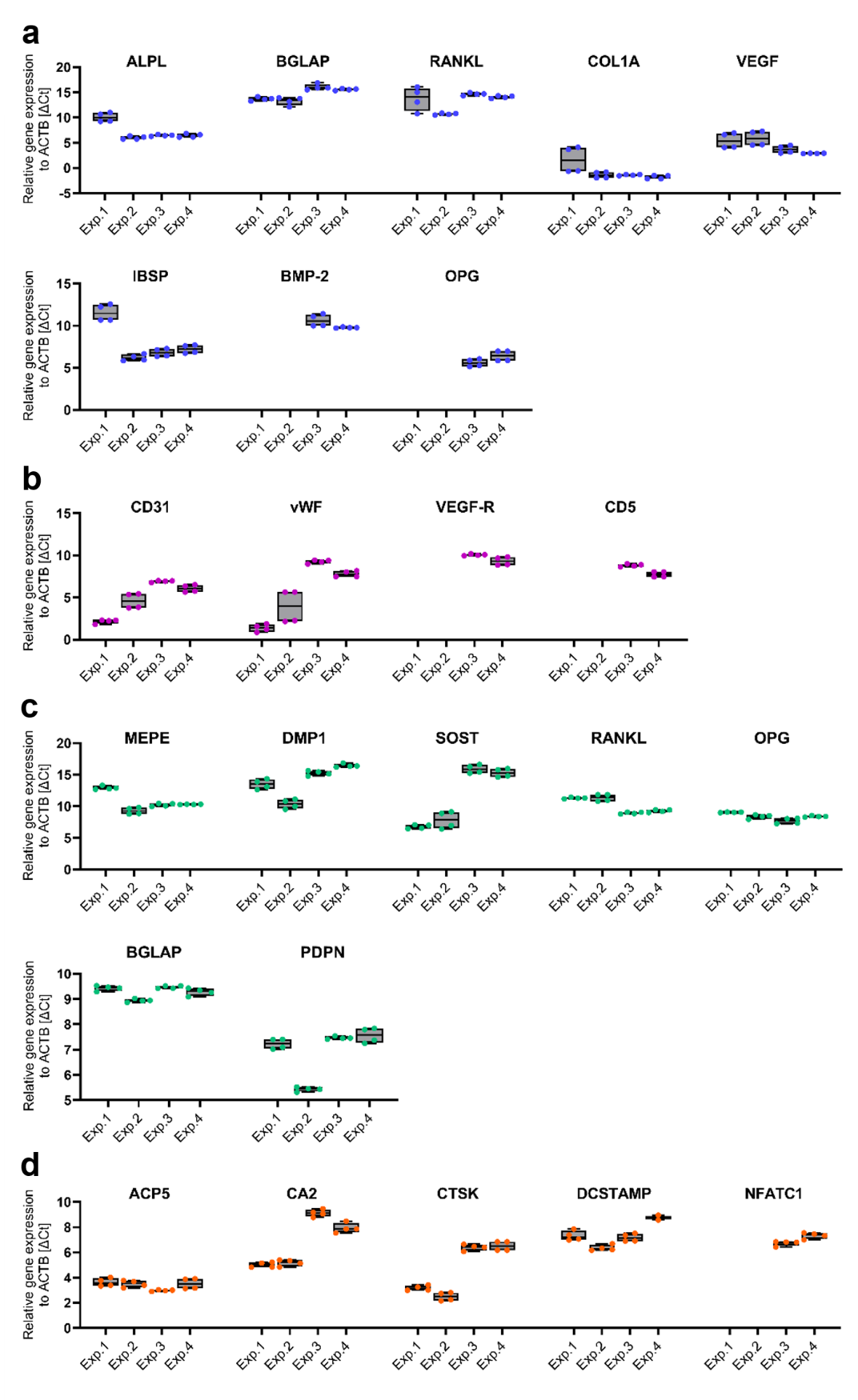


**Figure S2:** Gene expression of cell type specific markers in four quadruple culture experiments with different donor combinations under the defined control quadruple culture conditions (normoxic cultivation with VEGF + BMP-2 supplementation), presented as ΔCt. (a) OB marker, (b) Endothelial cell marker, (c) OCy marker, (d) OC marker. Each experiment *n*=4.
